# SSATNet: Spectral-spatial attention transformer for hyperspectral corn image classification

**DOI:** 10.3389/fpls.2024.1458978

**Published:** 2025-01-16

**Authors:** Bin Wang, Gongchao Chen, Juan Wen, Linfang Li, Songlin Jin, Yan Li, Ling Zhou, Weidong Zhang

**Affiliations:** ^1^ School of Life Sciences, Henan Institute of Science and Technology, Xinxiang, China; ^2^ School of Information Engineering, Henan Institute of Science and Technology, Xinxiang, China; ^3^ School of Art, Henan University of Economics and Law, Zhengzhou, China; ^4^ School of Software, Henan Institute of Science and Technology, Xinxiang, China

**Keywords:** corn identification, hyperspectral image classification, deep learning, morphology, image classification

## Abstract

Hyperspectral images are rich in spectral and spatial information, providing a detailed and comprehensive description of objects, which makes hyperspectral image analysis technology essential in intelligent agriculture. With various corn seed varieties exhibiting significant internal structural differences, accurate classification is crucial for planting, monitoring, and consumption. However, due to the large volume and complex features of hyperspectral corn image data, existing methods often fall short in feature extraction and utilization, leading to low classification accuracy. To address these issues, this paper proposes a spectral-spatial attention transformer network (SSATNet) for hyperspectral corn image classification. Specifically, SSATNet utilizes 3D and 2D convolutions to effectively extract local spatial, spectral, and textural features from the data while incorporating spectral and spatial morphological structures to understand the internal structure of the data better. Additionally, a transformer encoder with cross-attention extracts and refines feature information from a global perspective. Finally, a classifier generates the prediction results. Compared to existing state-of-the-art classification methods, our model performs better on the hyperspectral corn image dataset, demonstrating its effectiveness.

## 1 Introduction

Hyperspectral imaging technology comprehensively measures an object’s spectral properties by recording its absorption and reflection across various spectral bands (Li et al., 2024c; Zhang et al., 2024b; Li et al., 2024a). The resulting hyperspectral images, composed of multiple consecutive bands, are rich in feature information and can thoroughly reveal the nature of the object. This technology advances intelligent agriculture by utilizing the detailed feature information in hyperspectral images, thereby avoiding the destructive methods of traditional seed identification. Hyperspectral imaging has gradually been applied to intelligent agriculture, geological exploration, and medical treatment, offering new development opportunities and technical capabilities.

The increasing variety of corn seeds available in the market presents a significant challenge to the cereal farming industry, making the accurate identification of corn varieties especially crucial. Recently, researchers have been investigating hyperspectral image classification techniques using machine learning and deep learning approaches (Zhang et al., 2023c; Wu et al., 2022). Ahmad et al. (Ahmad et al., 2019). utilized a self-encoder paired with a multilayer extreme learning machine to mitigate high computational overhead and the Thuesian phenomenon in hyperspectral images, which improved the accuracy of hyperspectral image classification. Okwuashi et al. (Okwuashi and Ndehedehe, 2020) introduced a deep support vector machine algorithm incorporating four kernel functions and demonstrated its effectiveness in hyperspectral image classification using publicly available datasets. Zhang et al. (Zhang et al., 2020) employed a deep forest model with hyperspectral imaging to classify rice seeds with different levels of frost damage in small sample datasets. Su et al. (Su et al., 2022) introduced a new semi-supervised method for hyperspectral image classification that integrates normalized spectral clustering with kernel learning, effectively addressing the issues of relevant features appearing in non-adjacent regions and the lack of non-Euclidean spatial correlation. Jin et al. (Jin et al., 2023) developed a cost-sensitive K-neighborhood algorithm to reduce noise interference, enhance spatial information utilization, and achieve robust performance in hyperspectral wheat image classification. Farmonov et al. (Farmonov et al., 2023) employed wavelet transform for feature extraction, combined with random forests and support vector machine algorithms, to localize crops in farmland and classify crop hyperspectral images, playing a significant role in crop growth monitoring and harvest prediction. Sim et al. (Sim et al., 2024) combined machine learning algorithms with hyperspectral imaging for fast, non-destructive detection of coffee origin without sample processing. Wang et al. (Wang et al., 2024b) proposed a cross-domain few-shot learning strategy utilizing a two-branch domain adaptation technique to mitigate distortion caused by enforcing different domain alignments, achieving effective cross-domain transfer learning for low/high spatial resolution data. Although machine learning methods have demonstrated exemplary performance in hyperspectral image classification, their reliance on manual or semi-automatic feature extraction limits their potential. The emergence of deep learning methods has enabled the automatic extraction of spectral, spatial and spatial-spectral features from hyperspectral images, leading to significant advancements in this field.

Zhang et al. (Zhang et al., 2019) created a straightforward 1D convolutional capsule network to tackle the high dimensionality and limited labeled samples in hyperspectral images, achieving effective feature extraction and classification. Wang et al. (Wang et al., 2020) developed an end-to-end cubic convolutional neural network that integrates Principal Component Analysis with 1D convolution for efficient extraction of spatial and spectral features. Roy et al. (Roy et al., 2020) proposed an improved residual network using an adaptive spatial-spectral kernel with attention mechanisms, utilizing 3D convolutional kernels to simultaneously extract spatial and spectral features, achieving excellent classification results. Cui et al. (Cui et al., 2021) introduced a lightweight deep network using 3D deep convolution to classify hyperspectral images with fewer parameters and lower computational costs. Ortac et al. (Ortac and Ozcan, 2021) evaluated the performance of 1D, 2D, and 3D convolutions in hyperspectral image classification, demonstrating that 3D convolution offers superior feature extraction capabilities. Ghaderizadeh et al. (Ghaderizadeh et al., 2021) employed depth-separable and fast convolutional blocks in combination with 2D convolutional neural networks to effectively tackle data noise and insufficient training samples. Paoletti et al. (Paoletti et al., 2023a) proposed a channel attention mechanism to automatically design and optimize convolutional neural networks, reducing the computational burden in feature extraction while obtaining effective classification outcomes. Sun et al. (Sun et al., 2023) introduced an extensive kernel spatial-spectral attention network designed to efficiently leverage 3D spatial-spectral features, maintaining the 3D structure of hyperspectral images. Jia et al. (Jia et al., 2023) developed a structure-adaptive CNN for hyperspectral image classification, which employs structure-adaptive convolution and mean pooling to extract deep spectral, spatial, and geometric features from a uniform hyperpixel region. Gao et al. (Gao et al., 2023) designed a lightweight 3D-2D multigroup feature extraction module for hyperspectral image classification, which mitigates the loss of crucial details in single-scale feature extraction and the high computational expense of multiscale extraction. Zhang et al. (Zhang et al., 2023b) introduced a method combining 3D and 2D convolution to fully utilize the spatial, texture and spectral features of hyperspectral data for the task of identifying wheat varieties. In conclusion, while 2D and 3D convolutions effectively capture spectral and spatial features from hyperspectral data, traditional convolutional neural networks are limited by high computational complexity and insufficient feature utilization, impacting their classification performance.

Inspired by (Vaswani et al., 2017), researchers have suggested a Transformer-based network model for image classification (Zhang et al., 2024a). Hong et al. (Hong et al., 2021) effectively classified hyperspectral remote sensing images by leveraging spectral local sequence information from neighboring bands, considering the temporal properties, and designing cross-layer skipping connections combined with the Transformer structure. Roy et al. (Roy et al., 2021) introduced an innovative end-to-end deep learning framework, using spectral and spatial morphological blocks for nonlinear transformations in feature extraction. Yang et al. (Yang et al., 2022) integrated convolutional operations into the Transformer structure to capture local spatial context and subtle spectral differences, fully utilizing the sequence attributes of spectral features. Sun et al. (Sun et al., 2022b) developed a spatial-spectral feature tokenization converter to capture both spectral-spatial and high-level semantic features, achieving hyperspectral image classification through a feature transformation module, a feature extraction module, and a sample label learning module. Kumar et al. (Kumar et al., 2022) developed a novel morphology-expanding convolutional neural network that connects the morphological feature domain with the original hyperspectral data, reducing computational complexity and achieving good classification results. Peng et al. (Peng et al., 2022) developed a two-branch spectral-spatial converter with cross-attention, using spatial sequences to extract spectral features and capture deep spatial information to establish interrelationships among spectral sequences. Tang et al. (Tang et al., 2023) introduced a dual-attention Transformer encoder based on the Transformer backbone network for hyperspectral image classification, effectively extracting global dependencies and local spatial information between spectral bands. Qi et al. (Qi et al., 2023a) embedded 3D convolution in a two-branch Transformer structure to capture globally and locally correlated spectral-spatial domain features, demonstrating good performance for hyperspectral image classification through validation. Qiu et al. (Qiu et al., 2023) proposed a cross-channel dynamic spectral-spatial fusion Transformer capable of extracting multi-channel and multi-scale features, using multi-head self-attention to extract cross-channel global features and enhancing spatial-spectral joint features for hyperspectral image classification. Sun et al. (Sun et al., 2024) converted the spatial-spectral features into a memory marker storing *a priori* knowledge into an in-memory tagger, using a memory-enhanced Transformer encoder for the hyperspectral image classification task. Ahmad et al. (Ahmad et al., 2024) designed a Transformer-based network for hyperspectral image classification by combining wavelet transform with downsampling. The wavelet transform performs reversible downsampling, enabling attentional learning while preserving data integrity. Based on these studies, we propose utilizing a combination of 2D-3D convolution and Transformer, leveraging spectral-spatial morphological features to identify hyperspectral corn seed varieties. The contributions of this paper can be summarized as follows:

- We developed a 3D-2D convolutional cascade structure that autonomously extracts contextual features, reduces data complexity and efficiently captures high-level abstract features for integration into the Transformer architecture.
- We introduced a spectral-spatial morphology structure that employs expansion and erosion operations for spectral-spatial morphology convolution, enhancing the understanding of the data’s intrinsic properties.
- We employed a Transformer Encoder with CrossAttention to comprehensively extract and refine feature information from hyperspectral corn images on a global scale using the attention mechanism.

## 2 Related works

Currently, researchers have proposed a variety of methods for classifying hyperspectral remote sensing images and hyperspectral seed images. We classify these approaches into deep learning methods, machine learning methods and traditional methods. The deep learning methods are further divided into hybrid CNN-Transformer methods, Transformer-based methods, and CNN-based methods. Next, we overview and summarize these research outcomes.

**Traditional methods** for hyperspectral image classification primarily rely on analyzing physical and statistical features. These methods typically include spectral feature extraction, pixel-based classification, and target-based classification. For example, Cui et al. (Cui et al., 2020) introduced a super-pixel and multi-classifier fusion approach to tackle the challenges of limited labeled samples and substantial spectral variations. Similarly, Chen et al. (Chen et al., 2021a) introduced a feature extraction means that combines PCA and LBP, optimized using the Gray Wolf optimization algorithm for hyperspectral image classification. While these methods perform well for simpler classification tasks, their effectiveness diminishes when faced with complex backgrounds and highly mixed pixels.

**Machine learning methods** effectively classify hyperspectral images by learning the features of sample data. With the advancement of machine learning technology, researchers increasingly utilize machine learning algorithms for hyperspectral image classification. For example, Pham et al. (Pham and Liou, 2022) developed a push-sweep hyperspectral system using a support vector machine to date surface defects, addressing the problem of insufficient accuracy and speed in detecting date skin defects with traditional methods. Sun et al. (Sun et al., 2022a) constructed a network integrating multi-feature and multi-scale extraction with a swift and efficient kernel-extreme learning machine for rapid classification, significantly enhancing hyperspectral image classification accuracy. Wang et al. (Wang et al., 2023b) proposed a capsule vector neural network that combines capsule representation of vector neurons with an underlying fully convolutional network, achieving good classification performance with insufficient labeled samples. Compared to traditional methods, machine learning approaches handle high-dimensional data more effectively and achieve higher classification accuracy. However, these methods still rely on human-designed feature extraction and selection, preventing them from fully utilizing all the information in hyperspectral data.

**Deep learning methods** excel in hyperspectral image classification due to their automatic feature extraction and end-to-end learning capability (Zhang et al., 2024c; Hong et al., 2023). These methods can be categorized into hybrid CNN-Transformer methods, Transformer-based methods, and CNN-based methods.

CNN-based methods are designed to capture spectral and spatial features through convolutional layers specifically tailored for hyperspectral data, significantly improving classification performance (Wu et al., 2021). Yang et al. (Yang et al., 2021) introduced a spatial-spectral cross-attention network that suppresses redundant data bands and achieves robust, accurate classification. Yu et al. (Yu et al., 2021) developed a spectral-spatial dense convolutional neural network framework with a feedback attention mechanism to tackle issues of high complexity, information redundancy, and inefficient description, thereby improving classification efficiency and accuracy. Zheng et al. (Zheng et al., 2022) developed a rotationally invariant attention network for pixel feature class recognition, leveraging spectral features and spatial information. Paoletti et al. (Paoletti et al., 2023b) created a channel attention mechanism to automatically design and optimize a CNN, integrating 1D and spectral-spatial (3D) classifiers to process data from various perspectives while reducing computational overhead. Guo et al. (Guo et al., 2023) introduced a dual-view global spatial and spectral feature fusion network that efficiently extracts spectral-spatial features from hyperspectral images, accounting for global and local information.

Transformer-based methods excel at capturing long-range dependencies and complex features in hyperspectral images through a self-attention mechanism. Huang et al. (Huang et al., 2022) introduced a 3D swin transformer that captures rich spatial-spectral information, learns semantic representations from unlabeled data, and overcomes traditional methods’ limitations regarding receptive fields and labeling requirements. Yu et al. (Yu et al., 2022) proposed a multilevel spatial-spectral transformer network that processes hyperspectral images into sequences, addressing issues faced by CNN-based methods such as limited receptive fields, information loss in downsampling layers, and high computational resource consumption. Zhang et al. (Zhang et al., 2023d) developed a location-lightweight multi-head self-attention module and a channel-lightweight multi-head self-attention module, allowing each channel or pixel to associate with global information while reducing memory and computational burdens. Zhao et al. (Zhao et al., 2023) proposed an active learning hyperspectral image classification framework using an adaptive super-pixel segmentation and multi-attention transformer, achieving good classification performance with small sample sizes. Wang et al. (Wang et al., 2023a) introduced a trispectral image generation channel that converts hyperspectral images into high-quality trispectral images, mitigating the spatial variability problem caused by complex imaging conditions. Compared to CNNs, transformers have significant advantages in processing global and multi-scale features, allowing for better handling of global information in hyperspectral images.

Methods that hybrid CNN and Transformer aim to utilize the strengths of both to enhance hyperspectral image classification performance. These hybrid methods typically employ Transformers to capture global dependencies and CNNs to extract local spatial features. Zhang et al. (Zhang et al., 2022a) designed a dual-branch structure combining Transformer and CNN branches, effectively extracting both global hyperspectral features and local spectral-spatial features, resulting in high classification accuracy. Zhang et al. (Zhang et al., 2023a) proposed a network that integrates Transformer and multiple attention mechanisms, utilizing spatial and channel attention to focus on salient information, thereby enhancing spatial-spectral feature extraction and semantic understanding. Qi et al. (Qi et al., 2023b) introduced a global-local 3D convolutional Transformer network, embedding a dual-branch Transformer in 3D convolution to simultaneously capture global-local correlations across spatial and spectral domains, addressing the restricted receptive field issue of traditional CNNs. Xu et al. (Xu et al., 2024) proposed a two-branch convolutional Transformer network based on 3D CNN and an improved Transformer encoder, integrating spatial and local-global spectral features with lower computational complexity. Chen et al. (Chen et al., 2024) developed the TCCU-Net, a two-stream collaborative network that learns spatial, spectral, local and global information end-to-end for effective hyperspectral unmixing. This integration enables the model to leverage both spectral and spatial information from hyperspectral images more comprehensively, enhancing classification robustness and accuracy.

## 3 Methodology

**Figure 1:**
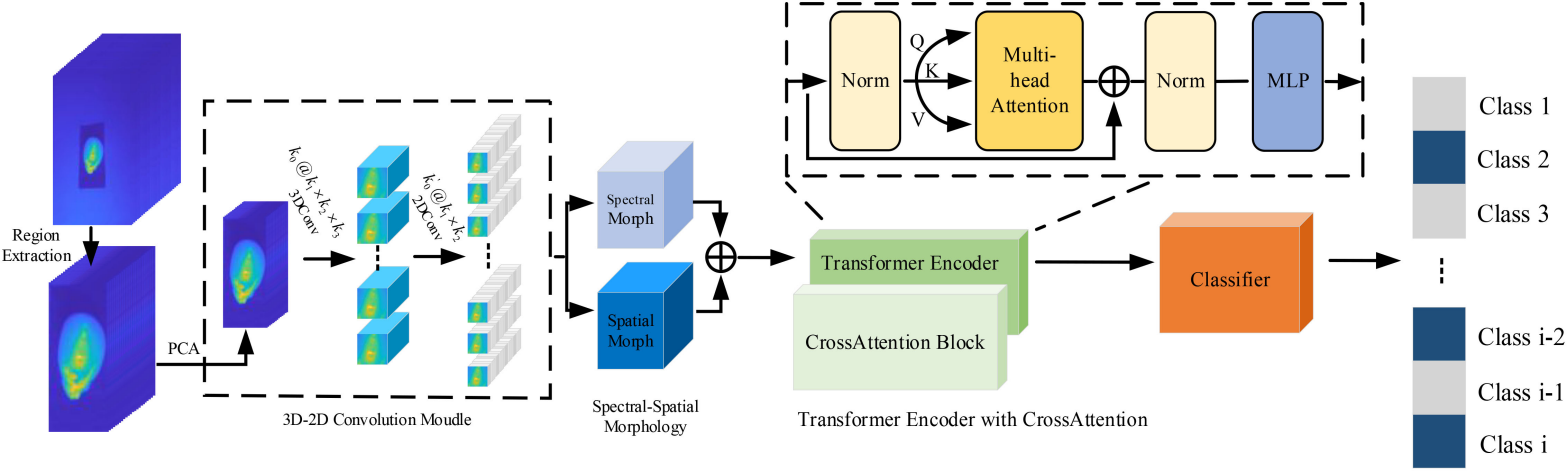
Flowchart of the spectral-spatial attention Transformer for hyperspectral corn image classification. Initially, the data are preprocessed with region of interest extraction and PCA dimensionality reduction. Subsequently, local spatial, spectral, and texture features are extracted using 2D and 3D convolutions. The spectral and spatial morphology modules further analyze the internal structure of the data. The Transformer encoder with cross-attention then extracts and refines the feature information from a global perspective. Finally, the classifier provides the prediction results.

The network flowchart of our proposed Spectral-Spatial Attention Transformer for hyperspectral corn image classification is shown in **Figure 1**. It contains 3D-2D Convolutional Module, Spectral-Spatial Morphology, Transformer Encoder with CrossAttention, and Classifier.

### 3.1 Motivation

With the development of intelligent agriculture, the integration of hyperspectral imaging technology and deep learning has gained widespread application in crop research, particularly in seed classification and identification. As a globally important food crop, the classification of corn seeds is significant for improving agricultural productivity and preserving crop genetic resources. Hyperspectral images can capture reflectance features at different wavelengths, providing researchers with rich spectral information for more precise seed classification and quality assessment (Chang et al., 2024).

In recent years, transformer models have emerged as popular in computer vision due to their powerful feature extraction and representation capabilities (Han et al., 2023; Li et al., 2024b). Compared to traditional convolutional neural networks, transformers are better at handling high-dimensional data and capturing long-range dependencies, which are crucial for extracting complex features from hyperspectral images. Additionally, the self-attention mechanism of Transformers enables the model to flexibly focus on important areas within the image, thereby enhancing classification accuracy. Consequently, choosing Transformer-based methods allows for more effective utilization of hyperspectral data, providing more reliable support for corn seed classification.

### 3.2 3D-2D convolution module

In hyperspectral image classification, effective feature extraction is vital for improving accuracy. Both 3D and 2D convolutions are widely used in this domain due to their unique advantages. 3D convolution simultaneously operates in spectral and spatial dimensions, capturing their correlation. Unlike traditional 2D or 1D convolutions, 3D convolution provides richer feature descriptions and retains more original spectral and spatial information, thus enhancing classification accuracy. It fully leverages the three-dimensional data structure of hyperspectral images, avoiding information loss or oversimplification. However, as network depth and input data size increase, the computational complexity and memory requirements of 3D convolution rise significantly, demanding higher hardware resources and more training time. 2D convolution, on the other hand, has lower computational complexity and high efficiency, as it operates on two-dimensional space (width and height). It effectively utilizes spatial and texture information, making it suitable for handling local features and texture details in hyperspectral images. Combining 3D and 2D convolutions can efficiently leverage the strengths to extract features from hyperspectral corn images. 3D convolution captures complex spectral-spatial relationships, while 2D convolution extracts local spatial features and texture information, maintaining computational efficiency. This combination optimizes feature extraction, leading to improved classification performance.

3D convolution is mainly used for three-dimensional data processing, extracting features by sliding a convolution kernel across the three dimensions of the input data. Suppose the input data is *I^D×H×W×C^*, where *C* is the number of channels, *W* is the width, *H* is the height, and *D* is the depth (spectral dimension).The dimensions of the 3D convolution kernel are $K_{d}\times K_{h}\times K_{w}\times C\times N$ , where *N* is the number of output channels (i.e., the number of convolution kernels), *C* is the number of input channels, $K_{w}$ is the size in the width direction, $K_{h}$ is the size in the height direction, and $K_{d}$ is the size of the convolution kernel in the depth direction. For an input tensor *I* and a convolution kernel *W*, the output tensor *Y* of the 3D convolution can be expressed as


(1) $$\begin{array}{c} Y\left(n,d,h,w\right)=\sum_{c=0}^{C-1}\sum_{k_{d}=0}^{K_{d}-1}\sum_{k_{h}=0}^{K_{h}-1}\sum_{k_{w}=0}^{K_{w}-1}I\left(c,d+k_{d},h+k_{h},w+k_{w}\right)\\ \times W\left(n,c,k_{d},k_{h},k_{w}\right)+b\left(n\right) \end{array}$$

where $I\left(c,d+k_{d},h+k_{h},w+k_{w}\right)$ is the value of the input tensor *I* at channel *c* and position $\left(d+k_{d},h+k_{h},w+k_{w}\right)$ . $W\left(n,c,k_{d},k_{h},k_{w}\right)$ represents the weight of the convolution kernel *W* at output channel *n* and input channel *c*, positioned at $\left(k_{d},k_{h},k_{w}\right)$ . b(n) is the bias term for each output channel *n* in the convolutional layers. It is initialized with random values (typically small values close to zero) and then adjusted during training via backpropagation. The gradient of the loss with respect to the bias is computed and used to update b(n) , just like the weights of the convolutional filters. This adjustment allows the model to shift the activations of each channel, enabling the network to adapt to various patterns in the data and improve its representation of features.

2D convolution is applied to 2D data processing, extracting features by sliding a convolution kernel (filter) across the two dimensions of the input data. Assuming the input data is *I^H×W×C^*, the 2D convolution kernel has dimensions $K_{h}\times K_{w}\times C\times N$ , with the parameter presentation consistent with that of 3D convolution. For an input tensor *I* and a convolution kernel *W*, the output tensor *Y* of the 2D convolution can be expressed as


(2) $$\begin{array}{c} Y\left(n,i,j\right)=\sum_{c=0}^{C-1}\sum_{k_{h}=0}^{K_{h}-1}\sum_{k_{w}=0}^{K_{w}-1}I\left(c,i+k_{h},j+k_{w}\right)\times W\left(n,c,k_{h},k_{w}\right)\\ +b\left(n\right) \end{array}$$

where $I\left(c,i+k_{h},j+k_{w}\right)$ is the value at position $\left(i+k_{h},j+k_{w}\right)$ in the input tensor *I* at channel *c*. $W\left(n,c,k_{h},k_{w}\right)$ represents the weight of the convolutional kernel *W* at position $\left(k_{h},k_{w}\right)$ for output channel *n* and input channel *c*.

### 3.3 Spectral-spatial morphology module

Hyperspectral images contain abundant textural, spatial, and spectral information. Morphology, a nonlinear image processing technique, is mainly used to analyze and manipulate the shape and structure of images. In hyperspectral image processing, morphological methods can effectively extract spatial and spectral features, enhancing the robustness and accuracy of image classification. Building on this, we integrate morphology with 2D convolution to locally manipulate images using structural elements, which can highlight or suppress specific shape features.

Spatial features can be extracted from each spectral band of a hyperspectral corn image through morphological operations like dilation and erosion. The dilation operation can emphasize the bright areas in the image and expand the edges of the target object, making the morphological features of the corn seed more pronounced. The computational expression for dilation is as


(3) $$D\left(I\right)=I⊕B=\;\cup_{b\in B}\left(I+b\right)$$

where *I* denotes the input image, *B* is the structural element (a small template used to detect the morphological features of the image), ⊕ stands for the dilation operation, $\cup_{b\in B}\left(\right)$ represents the union of all structural element positions to take the maximum value, and + denotes the pixel displacement operation. *b* influences the dilation and erosion operations. These operations involve shifting and adjusting the shape of features within the image, where *b* helps control the degree of expansion (dilation) or contraction (erosion). Like the convolutional biases, the values of *b* in these operations are also learned during training, refining the model’s ability to capture spatial relationships and remove irrelevant details in the data. Conversely, the erosion operation removes noise and small bright spots, resulting in a smoother and more uniform target area. The computational expression for erosion is as


(4) $$E\left(I\right)=I⊖B=\cap_{b\in B}\left(I-b\right)$$

where ⊖ denotes the erosion operation, $\cap_{b\in B}\left(\right)$ represents the intersection operation to take the minimum value for all structural element positions, and − indicates the negative displacement operation of pixels. Performing these operations on each spectral band extracts subtle spatial variations and enhances the representation of spatial features. Subsequently, these spatial features are combined with spectral features to fully utilize the spectral and spatial information in hyperspectral images. Specifically, we apply morphological operations to each spectral band to extract spatial features. These spatial features are merged with the original spectral information to construct high-dimensional feature vectors. This method preserves the spectral information of the hyperspectral image while enhancing the representation of spatial structure information. The feature extraction and classification effectiveness is further improved by integrating these morphological operations with 2D convolution. 2D convolution extracts local spatial features within each spectral band and enhances the representation of spatial information. These two convolutional operations complement each other, allowing the features, preprocessed through morphological operations, to be input into the convolutional neural network for more accurate classification.

The bias *b* in these equations plays a crucial role in adjusting the output activations, improving the feature extraction process. In the convolutional operations (Equations 1, 2), it allows the network to adapt to various activation patterns, enhancing the model’s ability to learn more complex relationships in the data. In the morphological operations (Equations 3, 4), it enhances spatial feature representation by refining the shapes and structures in the image. This combination of accurate feature extraction and refinement leads to better corn seeds classification performance.

By integrating morphological and convolutional techniques, we substantially enhance hyperspectral corn image classification accuracy and robustness. This combined approach boosts classification performance and improves resilience against complex backgrounds and noise.

### 3.4 Transformer encoder with CrossAttention module

**Figure 2:**
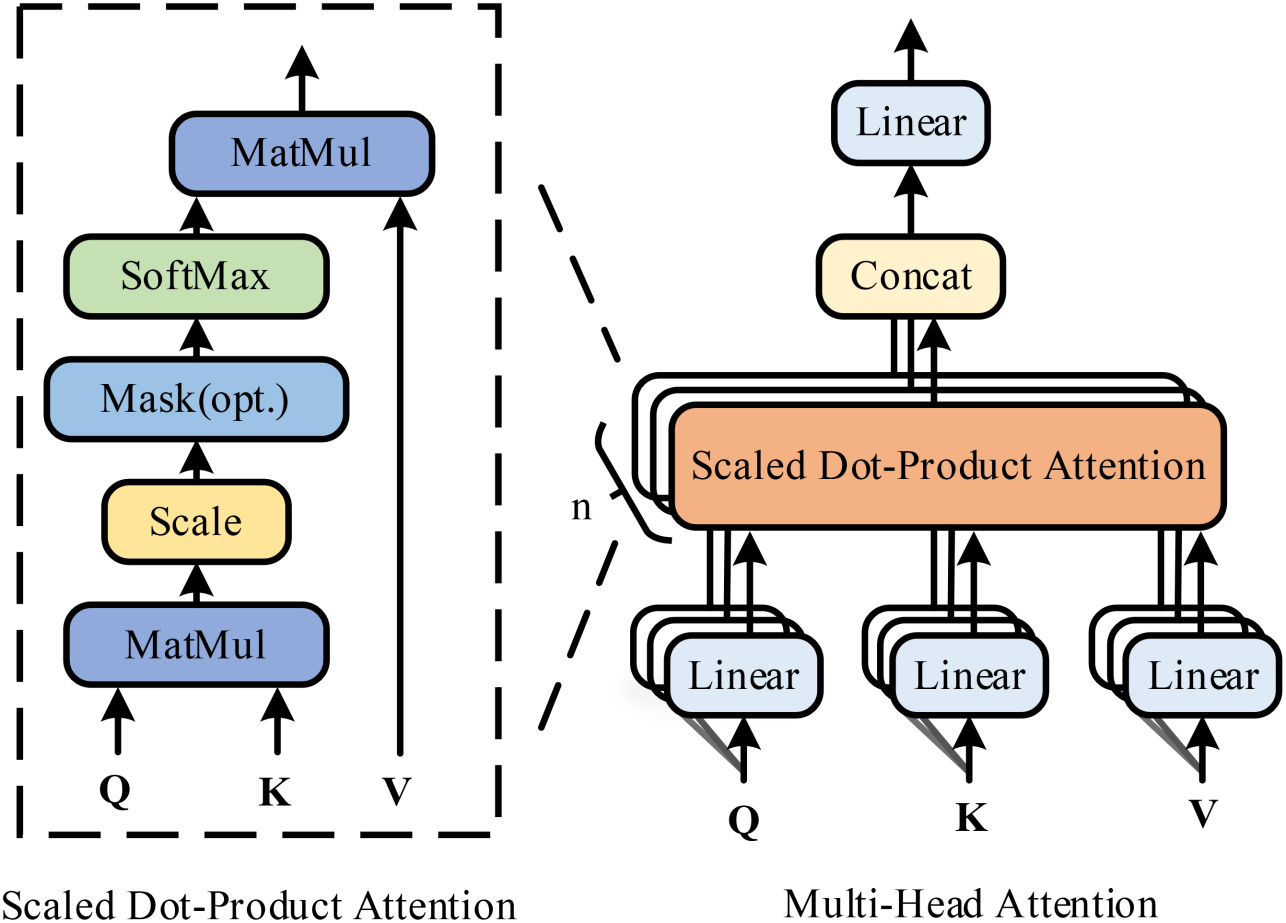
Diagram illustrating the structure of the multi-head attention mechanism and scaled dot-product attention.

The Transformer encoder enhances input data representation through a sophisticated attention module that captures dependencies among different parts of the input sequence. **Figure 2** depicts the detailed structure of this attention module, consisting of two primary components: multi-head self-attention and scaled dot-product attention.

Originally, the Transformer architecture was designed for natural language processing, particularly for handling sequence data, and it excels in this domain due to its multiple self-attention core blocks. Unlike conventional Convolutional Neural Networks and Recurrent Neural Networks, the Transformer exclusively utilizes the attention mechanism, enabling efficient capture of global dependencies in sequential data. The input sequence is initially converted into a fixed-dimensional vector representation via an embedding layer, with positional information preserved through positional encoding, which is generated by sine and cosine functions.

Each encoder layer includes multiple self-attention heads, each independently processing the input sequence to generate an attention representation, which is then concatenated and integrated through a linear transformation. The multi-head self-attention mechanism enables the model to attend to multiple parts of the input sequence simultaneously. Specifically, the input sequence is represented as a key (*K*), query (*Q*), and value (*V)*. Multiple sets of *Q*, *K*, and *V* are created through the linear projection of a learned weight matrix. Each set of *Q*, *K*, and *V* is passed to the scaled dot-product attention mechanism, where attention scores are calculated and applied to the values. The *Q* is multiplied by the transposed key $K^{T}$ to obtain the raw attention score, which is then divided by the square root of the key’s dimension, $\sqrt{d_{k}}$ , to maintain gradient stability. The computational process of self-attention can be summarized as


(5) $$\text{SA}=\text{Attention }\left(Q,K,V\right)=\text{softmax }\left(\frac{QK^{T}}{\sqrt{d_{K}}}\right)V$$

Through its unique multi-head self-attention mechanism and feed-forward neural network, the Transformer structure efficiently captures global dependencies and improves the classification accuracy of hyperspectral corn images.

### 3.5 Loss function

In this paper, we propose a method that combines spectral-spatial morphology with a 3D-2D convolutional Transformer network to classify hyperspectral corn images. This approach fully utilizes the spatial and spectral features of hyperspectral images. To optimize model performance, we employ the CrossEntropyLoss function.

The CrossEntropyLoss function is commonly used in classification tasks, especially for multi-class classification problems. It measures the discrepancy between the true category distribution and the predicted probability distribution by computing the negative log-likelihood between the actual labels and the predicted probabilities. This function ensures numerical stability by converting the output into a probability distribution using the Softmax function. Additionally, the gradient of the CrossEntropyLoss function is relatively easy to compute, facilitating the implementation of the back-propagation algorithm and model optimization. By directly quantifying the alignment between predicted probabilities and actual labels, it accurately reflects the performance of the classification model. Consequently, we apply the CrossEntropyLoss function to the hyperspectral corn image classification task. Its computational expression is as


(6) $$\text{CrossEntropyLoss}=-\sum_{i=0}^{N}\;y_{i}\text{ log }\left({\hat{y}}_{i}\right)$$

where $y_{i}$ represents the true label of the sample, *N* is the total number of samples, and ${\hat{y}}_{i}$ is the predicted probability from the model. The network model converts the output to a probability distribution using the Softmax function


(7) $${\hat{y}}_{i}=\frac{e^{z_{i}}}{\sum_{j}e^{z_{j}}}$$

where $z_{i}$ represents the linear output of the model. For a given category *c*, the true label $y_{c}=\;1$ while the labels for all other categories are 0. The predicted probability ${\hat{y}}_{i}$ corresponding to the true label $y_{i}$ is substituted into Equation 6, and the loss value for each sample is


(8) $$\text{Loss}=-\sum_{i}\;y_{i}\text{ log }\left({\hat{y}}_{i}\right)$$

By measuring the difference between actual and predicted labels and updating the model parameters through the backpropagation algorithm to minimize the loss, this approach effectively guides the model in learning to handle complex hyperspectral corn image features. Consequently, it improves both the classification accuracy and robustness.

## 4 Experiment and analysis

In this section, we will first discuss the dataset used, detail the specific implementation of SSATNet, and then present the evaluation metrics, multi-classification results, and ablation study.

### 4.1 Experimental dataset

**Figure 3:**
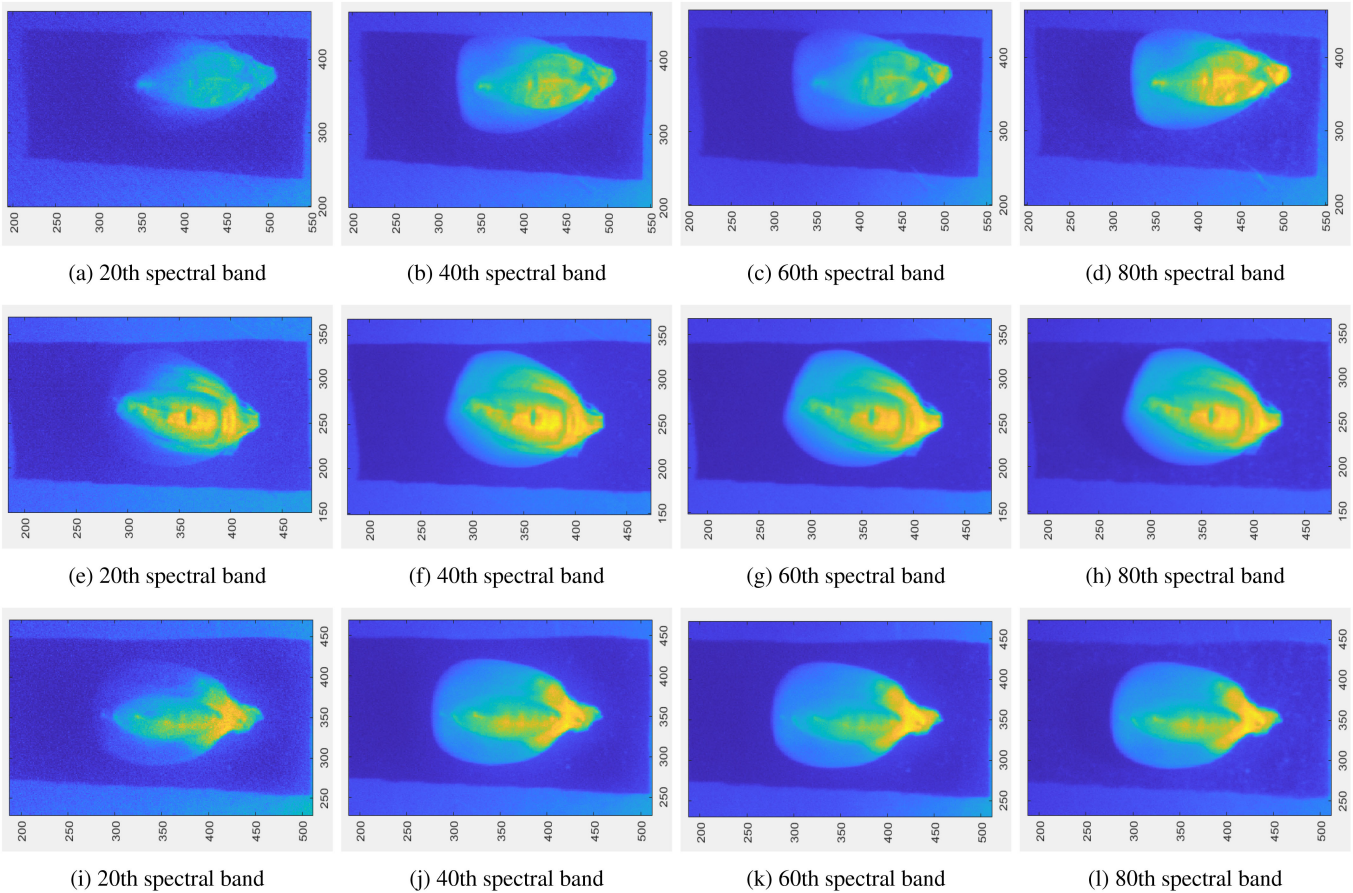
Randomly select a sample from three corn varieties, FengDa601 **(A–D)**, BaiYu818 **(E–H)**, and BaiYu833 **(I–L)**, and display their partial spectral bands.

To verify the effectiveness of the SSATNet, we utilized the hyperspectral corn image dataset from SSTNet (Zhang et al., 2022b). This dataset contains 10 corn varieties, each with 120 samples. The collected images cover a spectral range from 400 to 1000 nm, encompassing 128 bands. To reduce computational overhead and focus on retaining only the core area of the corn seeds, the collected raw data resolution of 696 × 520 was reduced to 210 × 200 for feature extraction. The corn seed images were sourced from planting areas in Henan Province, including varieties such as FengDa601, BaiYu9284, BaiYu8317, BaiYu918, BaiYu897, BaiYu879, BaiYu833, BaiYu818, BaiYu808, and BaiYu607. **Figure 3** shows different spectral band maps of a sample randomly selected from FengDa601, BaiYu818, and BaiYu833. This corn image dataset was obtained by contacting the authors.

### 4.2 Implementation details

The hyperspectral corn image dataset includes 10 varieties, totaling 1200 samples, divide into training and test sets in a 4:1 ratio. We conducted our experiments on a Windows 10 PC with an Intel^®^ Xeon^®^ Gold 5218 CPU @ 2.30GHz x64, an NVIDIA GeForce RTX 3090*2 graphics card, and 256 GB RAM. The Batch size is set to 16 for the training and 8 for the testing. We used Adamax as the optimizer with a learning rate of 0.01, an exponential decay rate of 0.9, a gradient squared moving average rate of 0.999, and 250 iterations. Additionally, we implemented a Dropout mechanism that randomly deactivates 10% of nodes, effectively preventing overfitting.

### 4.3 Evaluation metrics

**Figure 4:**
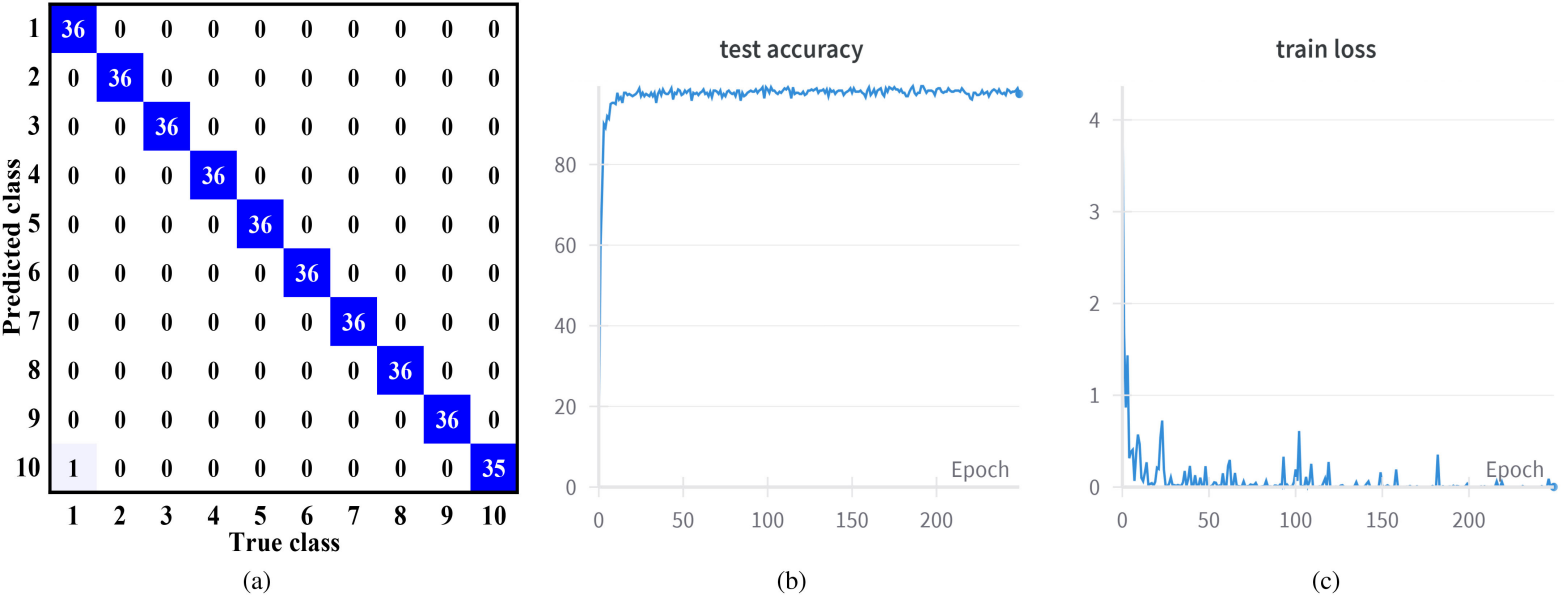
**(A)** The confusion matrix of our SSATNet classification results. **(B)** The results of one of the testing. **(C)** The results of one of the training.


To thoroughly assess the performance of our SSATNet in classifying hyperspectral corn images, we employ four standard evaluation metrics: F1-Score, Recall, Precision, and the Kappa coefficient(K_A_). Precision assesses the accuracy of the classification model by evaluating the proportion of instances predicted to be positive that are actually positive. There exists a trade-off between Precision and Recall; increasing Precision may lead to a decrease in Recall and vice versa. Therefore, the F1-Score, derived as the harmonic mean of Precision and Recall, is often used for a more balanced evaluation of model performance, and its calculation expression is shown in Equation 9. The K_A_ is a consistency test metric that evaluates the agreement between the classified image and the reference image in hyperspectral remote sensing classification tasks, providing a more comprehensive reflection of the overall classification accuracy. Higher scores in these four evaluation metrics indicate better model performance. **Figure 4** shows the confusion matrix of our model’s classification results for hyperspectral corn images and the results of one of the training and testing sessions.

(9) $$\text{F}1-\text{Score }=\;2\;▪\frac{\text{Precision }▪\text{ Recall}}{\text{Precision}+\text{Recall}}$$

### 4.4 Multi-classification results

Extensive experiments were performed to thoroughly test the generalization and effectiveness of our model for hyperspectral corn image classification. The comparison methods include KNN (Kumbure et al., 2020), SGD (Lei and Tang, 2021), RFA (Chen et al., 2021b), HybridNet (Roy et al., 2019), SSTNet (Zhang et al., 2022b), CTMixer (Zhang et al., 2022a), MSTNet (Yu et al., 2022), MATNet (Zhang et al., 2023a), and 3DCT (Wang et al., 2024a). The experimental results are presented in **Table 1**. The source code and parameters for the comparison methods were acquired from the original authors.

**Table 1:** Test results of various methods on the hyperspectral corn images dataset.

Models	Hyperspectral Corn images			
	Precision	Recall	F1-Score	K_A_
KNN (Kumbure et al., 2020)	96.12 ± 0.35	95.72 ± 0.32	95.90 ± 0.24	0.9675 ± 0.011
SGD (Lei and Tang, 2021)	96.98 ± 0.28	96.50 ± 0.18	96.70 ± 0.21	0.9721 ± 0.008
RFA (Chen et al., 2021b)	94.50 ± 0.40	94.10 ± 0.38	94.22 ± 0.39	0.9519 ± 0.009
HybridNet (Roy et al., 2019)	96.72 ± 0.30	96.44 ± 0.28	96.34 ± 0.21	0.9772 ± 0.007
SSTNet (Zhang et al., 2022b)	98.12 ± 0.18	97.78 ± 0.15	97.95 ± 0.17	0.9887 ± 0.005
CTMixer (Zhang et al., 2022a)	97.38 ± 0.33	97.75 ± 0.30	97.20 ± 0.32	0.9827 ± 0.008
MSTNet (Yu et al., 2022)	97.00 ± 0.38	96.95 ± 0.35	96.80 ± 0.36	0.9802 ± 0.009
MATNet (Zhang et al., 2023a)	98.27 ± 0.16	98.34 ± 0.14	98.25 ± 0.15	0.9930 ± 0.004
3DCT (Wang et al., 2024a)	98.30 ± 0.28	98.12 ± 0.25	98.19 ± 0.27	0.9928 ± 0.004
Our	**98.65 ± 0.18**	**98.57 ± 0.15**	**98.60 ± 0.17**	**0.9965 ± 0.003**

Optimal, bolded; Suboptimal, blue.

The results presented in **Table 1** demonstrate the performance of various methods on the hyperspectral corn images dataset. Traditional machine learning models such as KNN (Kumbure et al., 2020), RFA (Chen et al., 2021b), and SGD (Lei and Tang, 2021) show subpar performance across all evaluation metrics, with RFA (Chen et al., 2021b) performing the worst across all metrics. These traditional models, lacking nonlinear activation mechanisms, struggle to extract deep spectral-spatial features effectively. In contrast, HybridNet (Roy et al., 2019), SSTNet (Zhang et al., 2022b), and 3DCT (Wang et al., 2024a), which integrate 3D convolution, demonstrate superior results due to their ability to capture spectral and spatial features simultaneously. Models like CTMixer (Zhang et al., 2022a), MSTNet (Yu et al., 2022), and MATNet (Zhang et al., 2023a) further leverage the Transformer architecture to address the complex relationships inherent in hyperspectral data. Our proposed model, which combines convolutional networks with Transformers and incorporates a novel spectral-spatial attention mechanism, achieves the best overall performance across all metrics. The integration of local and global feature extraction methods allows our model to substantially improve Precision, Recall, F1-Score, and K_A_, surpassing existing state-of-the-art methods. These results validate the effectiveness of our design in capturing the complex spectral-spatial features of hyperspectral corn images and its superior ability to generalize to high-dimensional datasets.

### 4.5 Ablation study

To further evaluate the contribution of each module in SSATNet to the classification performance of hyperspectral corn seed images, we conducted ablation experiments on the dataset introduced by SSTNet (Zhang et al., 2022b). In these experiments, we systematically removed individual components of the network while retaining the remaining modules unchanged. Specifically, we excluded the following components: 1) the 3D convolution module (-w/o 3DConv); 2) the 2D convolution module (-w/o 2DConv); 3) the spectral morphology structure (-w/o SpectralMorph); and 4) the spatial morphology structure (-w/o SpatialMorph). The **Table 2** below illustrates the quantitative analysis metrics for each ablation experiment. The results demonstrate that the removal of the 3D convolution module leads to the most significant degradation in performance, underscoring its crucial role in capturing both spectral and spatial features in hyperspectral corn seed images. Without 3D convolution, the model’s ability to integrate spatial-spectral correlations is substantially weakened. Similarly, the removal of the 2D convolution module also causes a noticeable decline in performance, although to a lesser extent compared to the absence of 3D convolution. This is because 2D convolution primarily focuses on extracting local spatial features and refining feature representations. The exclusion of the spectral morphology structure results in performance degradation, highlighting its importance in enhancing spectral feature representation and managing the complex spectral relationships inherent in hyperspectral data. Likewise, the spatial morphology structure significantly contributes to the model’s performance by extracting and enhancing spatial features, enabling more accurate classification of corn seed images.

**Table 2:** Quantitative test results of ablation experiments.

Module	Precision	Recall	F1-Score	K_A_
-w/o 3DConv	86.42 ± 0.31	87.33 ± 0.29	87.05 ± 0.36	0.8768 ± 0.006
-w/o 2DConv	89.51 ± 0.25	90.35 ± 0.25	90.52 ± 0.29	0.9117 ± 0.004
-w/o SpectralMorph	93.65 ± 0.22	93.27 ± 0.19	93.86 ± 0.25	0.9408 ± 0.004
-w/o SpatialMorph	92.59 ± 0.20	92.69 ± 0.21	92.31 ± 0.21	0.9332 ± 0.005
SSATNet (full model)	**98.65 ± 0.18**	**98.57 ± 0.15**	**98.60 ± 0.17**	**0.9965 ± 0.003**

Optimal, bolded.

In summary, each module is crucial to the overall performance of SSATNet. The 3D convolution module provides the most significant enhancement to classification performance, followed by the spectral morphology structure and the spatial morphology structure. The 2D convolution module also provides substantial support in refining feature representation. Through the synergy of these modules, SSATNet excels in the hyperspectral corn seed classification task, demonstrating the effectiveness of its design.

## 5 Conclusion

In this paper, we propose the SSATNet method for non-destructive identification of hyperspectral corn varieties. First, we design a 3D-2D cascade structure to reduce image data complexity and effectively extract local feature information, facilitating the Transformer structure’s processing. Additionally, we introduce a spectral-spatial morphology structure combined with 2D convolution to perform expansion and erosion operations on the data, providing a deeper understanding of the data’s nature. Finally, we employ the Transformer structure to extract global feature information from hyperspectral corn images through the self-attention mechanism, achieving efficient capture of global dependencies between corn spectra. Ablation experiments highlight the effectiveness of each component of SSATNet in extracting features and classifying hyperspectral corn images. This method offers a new approach to non-destructive corn variety identification and significantly promotes the development of intelligent agriculture.

## Data Availability

The original contributions presented in the study are included in the article/supplementary material. Further inquiries can be directed to the corresponding author.
